# Deciphering the impact of nitrogen morphologies distribution on nitrogen and biomass accumulation in tobacco plants

**DOI:** 10.3389/fpls.2024.1377364

**Published:** 2024-07-01

**Authors:** Shichen Li, Tao Jiang, Waqar Ahmed, Yingfen Yang, Linyuan Yang, Tao Zhang, Fupeng Mei, Sulaiman Ali Alharbi, Qu Shan, Cuilian Guo, Zhengxiong Zhao

**Affiliations:** ^1^ Yunnan Agricultural University, Kunming, Yunnan, China; ^2^ Department of Botany & Microbiology College of Science, King Saud University, Riyadh, Saudi Arabia

**Keywords:** *Nicotiana tabacum*, nitrogen use efficiency, water-soluble nitrogen, non-protein nitrogen, SDS-insoluble nitrogen

## Abstract

**Background and aims:**

Nitrogen (N) distribution in plants is intricately linked to key physiological functions, including respiration, photosynthesis, structural development, and nitrogen storage. However, the specific effects of different N morphologies on N accumulation and plant growth are poorly understood. Our research specifically focused on determining how different N morphologies affect N absorption and biomass accumulation.

**Methods:**

This study elucidated the impact of different application rates (CK: 0 g N/plant; T1: 4 g N/plant; T2: 8 g N/plant) of N fertilizer on N and biomass accumulation in tobacco cultivars Hongda and K326 at different growth stages.

**Results:**

Our findings emphasize the critical role of N distribution in various plant parts, including leaves, stems, and roots, in determining the complex mechanisms of N and biomass accumulation in tobacco. We found that in relation to total N, a greater ratio of water-soluble N (*N*_w_) in leaves facilitated N accumulation in leaves. In contrast, an increased ratio of SDS (detergent)-insoluble N (*N*_in-SDS_) in leaves and non-protein N (*N*_np_) in roots hindered this increase. Additionally, our results indicate that a greater proportion of *N*_np_ in leaves has a negative impact on biomass accumulation in leaves. Furthermore, elevated levels of *N*_in-SDS_, *N*_w_, and *N*_np_ in roots, and *N*_np_ in leaves adversely affected biomass accumulation in tobacco leaves. The Hongda cultivar exhibited greater biomass and N accumulation abilities as compared to K326.

**Conclusions:**

Our findings highlight the significant role of distribution of N morphologies on plant growth, as well as N and biomass accumulation in tobacco plants. Understanding N distribution allows farmers to optimize N application, minimizing environmental losses and maximizing yield for specific cultivars. These insights advance sustainable agriculture by promoting efficient resource use and reducing environmental impact.

## 1 Introduction

Nitrogen (N) fertilizers contribute significantly to enhancing crop productivity and serve as a cornerstone for addressing global food security by substantially increasing crop yields (Cui et al., 2022). China is the world’s largest fertilizer producer and consumer, and from 1978 to 2006, its fertilizer input contributed 56.18% of the increase in grain yield (Wang et al., 2022b). Previous studies have highlighted the direct correlation between N application, N uptake, and improved plant growth parameters, including tobacco plant height, leaf number, leaf area, and stem diameter by 61.04% (Haghighi et al., 2011; Li et al., 2021). Despite these advances, a comprehensive understanding of the intricate distribution of N across various plant organs remains limited. This knowledge gap calls for a thorough investigation of the effects of diverse N allocation ratios on N and biomass accumulation in tobacco plants, particularly under different N conditions, growth stages, and cultivars.

In plants, water-soluble N (*N*_w_), SDS (detergent)-soluble N (*N*_s_), SDS (detergent)-insoluble N (*N*_in-SDS_), and non-protein N (*N*_np_) are commonly distributed distinct N morphologies (Liu et al., 2018). Each of these N morphologies plays a specific role within plant leaves; for example, *N*_w_ primarily facilitates photosynthesis and storage functions (Qiang et al., 2023), *N*_s_ is predominantly linked to photosynthesis including electron transfer and light capture (Viola et al., 2022), *N*_in-SDS_ is primarily present in cell wall proteins, which are assumed to contribute to the mechanical toughness of leaves and DNA integrity (Evans and Seemann, 1989; Takashima et al., 2004; Liu et al., 2018, 2023), and *N*_np_ is primarily involved in coordinating leaf expansion and photosynthetic activities (Liu et al., 2018).

Furthermore, the intricate relationship between N allocation and various agronomic measures, particularly N application, has been highlighted as the primary determinant of N distribution in leaves (Tian et al., 2023). High N application has been linked to increased *N*_s_ and *N*_w_ distributions in soybean and oilseed rape plants, which improves electron transfer, light capture, and overall plant growth (Qiang et al., 2023). It has been reported that increased N application could enhance the light-capturing ability of *Panax notoginseng* by optimizing the distribution of *N*_s_ (Cun et al., 2021). Comprehensive research is crucial for fully understanding the implications of N allocation across various plant organs, particularly at different growth stages and among different cultivars.

The influence of N morphology varied significantly across different growth stages. In earlier growth stages, a greater ratio of *N*_np_ has been observed to promote leaf development, whereas this ratio decreases during later growth stages when leaf expansion ceases (Liu et al., 2018). Qu et al. (2022) highlighted a decline in *N*_s_ and *N*_in-SDS_ during the flowering to early fruiting stages compared to the seedling stages of cucumbers, indicating a dynamic beneficial impact of these N morphologies during the seedling stages, but adverse effects during the early fruiting stages. Similarly, N allocation also determined the distinct characteristics of cultivars; among different cultivars of pecans, the greater ratio of *N*_s_ and *N*_w_ in the cultivar YLC35 promoted greater photosynthetic N-use efficiency (Xu et al., 2022), which benefited the growth of plants (Qiang et al., 2023). Conversely, a greater ratio of *N*_in-SDS_ in two different N environments adversely affected photosynthesis and respiration abilities in two canola plant cultivars (Liu et al., 2023). Our previous research also indicated that greater proportions of *N*_w_ and *N*_s_ can enhance N use efficiency, thereby benefiting both biomass and N accumulation (Li et al., 2023).

Understanding N accumulation and growth in tobacco is of utmost importance, considering its significant role in the rural economy of China (Sun et al., 2020). The present study primarily focused on N allocation in leaves, with limited insights into N distribution across different plant organs, morphological variations, and implications for crop growth and development under varying N conditions, growth stages, and cultivars (Hongda and K326). However, long-term unscientific fertilization not only affects yield but also seriously impacts the fragile ecological environment. This study aimed to bridge the existing knowledge gaps by investigating the relationships between N allocation and N and biomass accumulation across different plant organs in flue-cured tobacco cultivars at different growth stages (with a particular emphasis on the Hongda variety, which consistently accumulates more N and biomass under the same N application) under various treatments and at different growth stages. This study aimed to provide valuable insights for optimizing N fertilizer application, producing high-yield flue-cured tobacco leaves and reducing the ecological footprint.

## 2 Materials and methods

### 2.1 Experimental site

The study was conducted during two growing seasons from May to September 2021 and 2022 in Yongping County, Dali Prefecture (25.6065°N, 100.2676°E), Yunnan, China. The area receives an average annual precipitation of approximately 919.0 mm and an average temperature of 16.7°C. The soil at the research site was classified as loam and had the following basic physical and chemical properties: 36.58 g kg^−1^ organic matter, 1.95 g kg^−1^ total nitrogen, 0.82 g kg^−1^ total phosphorus, 17.88 g kg^−1^ total potassium, 233.10 mg kg^−1^ available nitrogen, 49.34 mg kg^−1^ available phosphorus, and 236.00 mg kg^−1^ available potassium.

### 2.2 Experimental design and conditions

In an open-field pot experiment, we aimed to assess the impact of different N fertilizer application rates on N efficiency in two flue-cured tobacco cultivars (Hongda and K326). Seedlings of the tobacco cultivars Hongda and K326 were transplanted into pots (25 cm × 24 cm), each containing 15 kg of loam soil. To ensure adequate soil moisture, each pot was irrigated with 1,000 mL of water thrice a week. Pots were strategically positioned in the field, maintaining a plant × row spacing of approximately 110 cm × 55 cm, which is consistent with a planting density of 16,500 plants per hectare (Cai et al., 2021). Each treatment consisted of three biological replicates, with 30 plants per cultivar within each replicate. Experiments were performed under three different application rates of N fertilizer (**Table 1**). Fertilizers were applied as base and top dressings according to standards for tobacco production (Ahmed et al., 2022). At the time of transplantation, the following percentages of fertilizer were added: 80% N, 100% P, and 80% K. Twenty-one days after transplantation, the remaining 20% of the estimated N and K were applied as a top dressing. All management practices were performed according to the guidelines outlined in China’s National Standards for the Tobacco Industry (Li et al., 2022).

**Table 1:** Experimental conditions under different application rates of nitrogen fertilizer.

Treatments	Cultivars	N application per plant	P application per plant	K application per plant
CK	Hongda	No N fertilizer	2.5 g	10 g
	K326	No N fertilizer	2.5 g	10 g
T1	Hongda	4 g	2.5 g	10 g
	K326	4 g	2.5 g	10 g
T2	Hongda	8 g	2.5 g	10 g
	K326	8 g	2.5 g	10 g

N, nitrogen; P, phosphorus; K, potassium.

### 2.3 Harvesting and sample analysis

Samples were collected at 25, 50, 75, 100, and 125 days after transplantation of tobacco seedlings. After being harvested, the tobacco plants were divided into five distinct parts: roots, stems, lower leaves, middle leaves, and upper leaves. Three tobacco plants per replicate were uprooted for each treatment at each growth stage. To remove excess soil, the plants were thoroughly rinsed with running water, as described by Hu et al. (2021). The fresh and dry weights of each plant part were recorded before and after curing. To ensure consistency, each plant part was dried at 105°C for 30 min, followed by drying at 75°C for 72 h as part of the curing process. Each dried plant part was sieved through a 2-mm mesh sieve before being crushed and digested with H_2_SO_4_-H_2_O_2_. According to the method outlined by Liu et al. (2018), the total N content of the digested plant material was determined through continuous flow analysis using an AA3 instrument (Seal Analytical Inc., Southampton, UK).

### 2.4 Nitrogen morphologies in tobacco

The N morphologies were assessed in frozen samples according to Takashima et al. (2004). Nitrogen was divided into four morphologies: water-soluble (*N*_w_), non-protein nitrogen (*N*_np_), SDS (detergent)-soluble nitrogen (*N*_s_), and SDS (detergent)-insoluble nitrogen (*N*_in-SDS_). One gram of each plant part, including leaves, roots, and stems, was cryogenically frozen in liquid N and subsequently homogenized with 1 mL of 100 mM sodium phosphate buffer [comprising 2 mM MgCl_2_, 0.4 M d-sorbitol, 5 mM dithiothreitol (DTT), 5 mM iodoacetate, 10 mM NaCl, and 5 mM phenylmethylsulfonyl fluoride]. The supernatant *N*_w_ was separated by centrifuging at 12,000 × *g* at 4°C for 10 min. The residual samples were dissolved in 1 mL of phosphate buffer (containing 3% SDS) for 5 min at 90°C and then centrifuged at 5,500 × *g* for 8 min to collect the supernatant as *N*_s_. The *N*_in-SDS_ residues were purified by washing with anhydrous ethanol (20 mL) and filtered through medium-speed quantitative filter paper. The supernatant was mixed with an equal volume of 20% trichloroacetic acid, filtered through medium-speed quantitative filter paper, and thoroughly rinsed with anhydrous ethanol (20 mL) to denature the N compounds. Three distinct N morphologies were determined from the residue on the quantitative filter paper after natural air-drying and subsequent digestion using H_2_SO_4_-H_2_O_2_ following Thomas’s method (Thomas et al., 1967). The N content of the digested solution was determined using continuous flow analysis (AA3; Seal Analytical Inc., Southampton, UK). The *N*_np_ content was calculated by subtracting the values of *N*_w_, *N*_s_, and *N*_in-SDS_ from the total N content. Percentage of each N morphologies in each organs were calculated using the following equation:


$$\begin{array}{c} Each\;N\;morphol\text{og}ies\;in\;leaves\;\left(\%\right)\\ =\frac{The\;content\;of\;each\;N\;morphologies\;in\;leaves\;*\;Biomass\;of\;leaves}{The\;total\;nitrogen\;accumulation\;of\;whole\;plant\;\left(sum\;of\;leaves,\;stems\;and\;roots\right)}*100\% \end{array}$$

The calculation of N morphology in stems and roots was similar to that for leaves.

### 2.5 Statistical analysis

The data were statistically analyzed using SPSS (version 23.0; Armonk, NY, USA) for *Z* score, curve fitting analysis, and analysis of variance (two-way ANOVA). To evaluate significant differences between treatments, the least significant difference (LSD) test was applied, with significance set at *p* < 0.05. Graphical representations were created using OriginLab Version 2022 (Northampton, MA, USA) and further refined and compiled using Adobe Illustrator Version 2019 (San Jose, CA, USA).

## 3 Results

### 3.1 Nitrogen accumulation in the leaves of tobacco cultivars at different growth stages under different rates of N application

**Figure 1:**
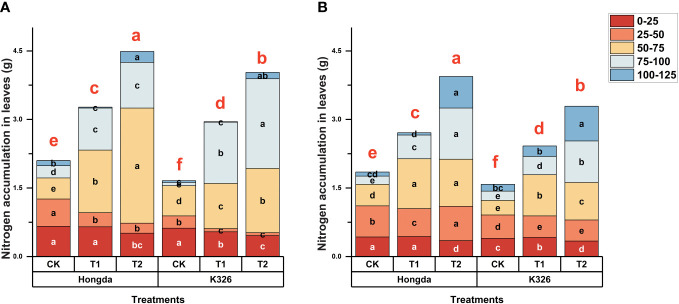
Stacked bar charts illustrating nitrogen accumulation in tobacco plants at different time intervals (0–25 d, 25–50 d, 50–75 d, 75–100 d, and 100–125 d) after transplantation. Here, control (CK): no N fertilizer (0 g/plant), medium (T1): 4 g/plant of pure N, and high (T2): 8 g/plant of pure N. The different colors represent the N accumulation at different growth stages. The total height of the bar demonstrates the difference in N accumulation at 125 d after transplanting. Lowercase letters within the same growth stage indicate significant differences among treatments according to the least significant difference test (LSD, *p* < 0.05). Specifically, after 125 days of transplantation, the significant differences among treatments are indicated by red lowercase letters above the bar chart (LSD; *p* < 0.05). **(A, B)** represent the results for 2021 and 2022, respectively.

**Figure 1** shows that higher levels of N application led to increased N accumulation in tobacco leaves during 125 d of growth after transplantation in 2021 (**Figure 1A**) and 2022 (**Figure 1B**). During the initial 50 d following transplantation, CK exhibited 18.80% and 32.38% greater N accumulation in tobacco leaves than treatments T1 and T2, respectively, indicating that lower N application resulted in greater N accumulation during the early growth stages. However, at 75 d, higher levels of N application increased N accumulation in tobacco leaves, highlighting the dynamic response of tobacco plants to varying N levels at different growth stages. Additionally, at 125 d, N accumulation in CK was 63.36% and 45.65% lower than N accumulation in T1 and T2, respectively. The Hongda cultivar, known for its superior N efficiency, displayed 15.25% higher N accumulation in leaves than the K326 cultivar.

### 3.2 Biomass accumulation in tobacco leaves at different growth stages under different rates of N application

**Figure 2:**
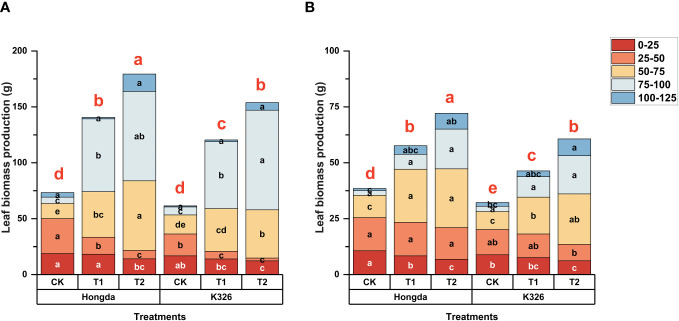
Stacked bar charts illustrating biomass accumulation in tobacco plants at different time intervals (0–25 d, 25–50 d, 50–75 d, 75–100 d, and 100–125 d) after transplantation. Here, control (CK): no N fertilizer (0 g/plant), medium (T1): 4 g/plant of pure N, and high (T2): 8 g/plant of pure N. The different colors represent the N accumulation at different growth stages. The total height of the bar demonstrates the difference in N accumulation at 125 d after transplantation. Lowercase letters within the same growth stage indicate significant differences among treatments according to the least significant difference test (LSD, *p* < 0.05). Specifically, after 125 days of transplantation, the significant differences among treatments are indicated by red lowercase letters above the bar chart (LSD; *p* < 0.05). **(A, B)** represent the results for 2021 and 2022, respectively.

**Figure 2** shows a strong correlation between N application and biomass accumulation in tobacco leaves in 2021 (**Figure 2A**) and 2022 (**Figure 2B**). This trend is closely correlated with the pattern observed for N accumulation in tobacco leaves. Higher levels of N application corresponded to increased tobacco leaf biomass, particularly at 125 d after transplantation. During the initial stages of tobacco growth (0–50 days after transplanting), CK exhibited 37.89% and 85.93% more biomass accumulation compared to T1 and T2 treatments, respectively. After 75 d of transplantation, higher levels of N application led to increased tobacco leaf biomass production. However after 125 d of transplantation, CK accumulated 56.25% and 44.07% lower biomass than T1 and T2, respectively. Moreover, the Hongda cultivar exhibited 18.23% greater tobacco leaf biomass production than the K326 cultivar under similar treatment conditions in both 2021 and 2022.

### 3.3 Variations in allocation ratios of different nitrogen morphologies in tobacco leaves at different growth stages under different N fertilizer treatments

The different application levels of N fertilizer at various growth stages across the two tobacco cultivars differently induced the distribution of various nitrogen morphologies within the tobacco plant parts (**Table 2**). A notable trend was observed throughout an extensive 2-year study: *N*_in-SDS_ levels in tobacco leaves decreased with increased N application. Conversely, there was an overall increase in the levels of *N*_w_, *N*_s_, and *N*_np_ in the tobacco leaves in response to augmented N application. As the growth of the tobacco plants progressed, a noticeable decrease in the ratio of *N*_in-SDS_ was observed, whereas the *N*_s_ and *N*_np_ ratios consistently increased. The Hongda cultivar exhibited greater ratios of *N*_w_ and *N*_s_ in leaves as compared to K326, but showed lower levels of *N*_np_.

**Table 2:** Allocation ratios of different nitrogen morphologies in tobacco leaves at different growth stages under various treatments.

Days after transplanting	Cultivars	Treatments	2021				2022			
			*N*_in-SDS_ (%)	*N*_w_ (%)	*N*_s_ (%)	*N*_np_ (%)	*N*_in-SDS_ (%)	*N*_w_ (%)	*N*_s_ (%)	*N*_np_ (%)
25	Hongda	CK	4.32a	53.37a	18.58b	13.76bc	12.80a	19.81a	37.74a	1.32d
		T1	10.81a	47.89a	26.24a	5.77c	10.39a	17.58a	34.50a	11.32c
		T2	6.50a	49.00a	19.93b	17.84bc	10.98a	15.50a	25.99b	22.74b
	K326	CK	15.30a	20.54b	12.31d	41.30a	13.92a	16.44a	35.30a	8.19cd
		T1	14.44a	32.22b	16.88bc	26.40b	11.79a	9.31b	32.39ab	24.35b
		T2	9.56a	20.47b	13.42cd	45.75a	11.09a	9.49b	19.02c	38.63a
50	Hongda	CK	7.22b	40.93abc	12.73a	16.19ab	14.56bc	21.85a	20.96bc	7.20c
		T1	6.90b	54.40a	10.73a	4.11ab	14.10bc	24.64a	23.18ab	3.85c
		T2	11.09ab	37.02bc	12.28a	21.94a	12.80c	22.14a	26.36a	6.53c
		CK	15.92a	41.21abc	13.48a	2.90b	12.51c	16.10b	18.13cd	18.22a
		T1	13.15ab	51.34ab	11.98a	1.68b	16.69b	22.11a	18.73bcd	13.86ab
		T2	10.37ab	37.03c	12.67a	16.75a	25.06a	16.98b	16.06d	8.68bc
75	Hongda	CK	2.49cd	27.77c	13.87b	8.29bc	17.75bc	20.44a	19.80a	11.57b
		T1	1.14d	37.59b	20.74a	1.29c	19.38b	17.46bc	18.41ab	7.43b
		T2	6.83bc	45.22a	12.98b	17.30b	7.62d	25.57a	20.23a	8.17b
	K326	CK	14.02a	24.23c	11.16bc	3.04bc	23.22a	18.32ab	16.20ab	9.43b
		T1	11.59ab	30.35c	14.63b	3.99bc	18.43bc	15.12c	16.33ab	20.37a
		T2	8.52ab	26.21c	8.32c	30.50a	16.43c	19.12ab	14.74b	14.00b
100	Hongda	CK	8.60a	21.53c	14.58a	4.99a	22.14b	19.57ab	15.66b	9.83b
		T1	8.79a	34.39ab	15.65a	4.22a	15.30c	21.88a	15.55b	8.43b
		T2	7.68a	38.40a	14.22a	11.14a	14.43c	22.83a	20.60a	2.94c
	K326	CK	8.96a	19.99c	12.58a	3.69a	22.41b	15.40c	13.33c	14.63a
		T1	10.86a	28.39bc	13.93a	8.52a	32.78a	16.34bc	13.42c	4.28c
		T2	8.13a	31.60ab	14.67a	13.09a	24.12b	20.35a	13.73c	3.85c

Control (CK): no N fertilizer (0 g/plant), medium (T1): 4 g/plant of pure N, and high (T2): 8 g/plant of pure N. Different lowercase letters within a column show the significant difference among treatments according to the least significant difference test (LSD; p < 0.05). N_in-SDS_, SDS-insoluble nitrogen; N_s_, SDS-soluble nitrogen; N_w_, water-soluble nitrogen; and N_np_, non-protein nitrogen.

### 3.4 Variations in the ratios of different nitrogen morphologies in tobacco roots at various growth stages under different N fertilizer treatments

Different N application levels, growth stages, and cultivars led to distinct alterations in the distribution of nitrogen morphologies within tobacco roots (**Table 3**). Over 2 years, a collective decrease in the ratios of *N*_w_, *N*_s_, and *N*_np_ in tobacco roots was observed in response to increasing N application. Simultaneously, the proportion of *N*_in-SDS_ decreased as the tobacco plants grew. In comparison to K326, the roots of the Hongda cultivar contained lower proportions of both *N*_in-SDS_ and *N*_np_.

**Table 3:** Allocation ratios of different nitrogen morphologies in tobacco roots at different growth stages under various treatments.

Days after transplanting	Cultivars	Treatments	2021				2022			
			*N*_in-SDS_ (%)	*N*_w_ (%)	*N*_s_ (%)	*N*_np_ (%)	*N*_in-SDS_ (%)	*N*_w_ (%)	*N*_s_ (%)	*N*_np_ (%)
25	Hongda	CK	0.72ab	3.35a	0.94a	1.40b	0.35b	6.86a	4.49a	5.16c
		T1	0.69ab	2.70ab	0.69abc	1.16b	0.38b	4.23c	3.34b	5.58c
		T2	0.33b	0.83d	0.37c	2.28ab	0.46b	5.70b	3.66ab	1.88d
	K326	CK	0.76ab	1.89bc	0.55bc	2.31ab	0.40b	4.31c	2.09c	10.20a
		T1	0.43ab	1.41cd	0.54bc	3.34a	0.39b	2.21d	1.21c	9.01ab
		T2	0.86a	2.86a	0.70ab	2.16ab	1.41a	3.51c	1.22c	7.64b
50	Hongda	CK	3.29b	4.30b	2.65a	0.86c	0.12d	5.25b	3.29a	0.23e
		T1	2.11d	3.17c	0.84c	1.86b	0.14cd	5.54a	2.14c	0.18e
		T2	2.36cd	3.28bc	1.84ab	0.32c	0.27bcd	1.55f	2.50b	0.39d
	K326	CK	5.23a	6.15a	2.47a	0.82c	0.32bc	2.89d	1.27d	5.22a
		T1	3.06bc	4.49b	1.87ab	1.75b	0.40b	3.54c	0.62e	4.09b
		T2	3.29b	3.77bc	1.44bc	4.25a	4.64a	2.34e	2.14c	0.92c
75	Hongda	CK	3.47ab	10.33ab	4.68a	9.08a	0.88c	4.18a	2.49a	0.20c
		T1	3.96a	9.04abc	3.48bc	4.29b	0.58cd	3.03b	1.62b	0.11c
		T2	1.52d	5.21c	1.24e	2.63b	0.08d	1.48d	0.84c	0.14c
	K326	CK	3.88ab	12.62a	3.82b	9.46a	3.64a	2.51c	0.79c	1.12a
		T1	2.32cd	8.18bc	2.76d	8.97a	2.16b	1.88d	0.90c	1.03a
		T2	2.70bc	8.77abc	2.99cd	5.81ab	3.20a	1.78d	0.54d	0.56b
100	Hongda	CK	2.09bc	9.42ab	2.84a	11.19a	1.04d	3.37a	2.33b	0.24bc
		T1	2.76b	9.67a	3.21b	1.35b	1.20d	3.52a	2.86a	0.08c
		T2	1.39c	4.02c	1.58c	1.22b	0.33e	2.11b	1.12d	0.17bc
	K326	CK	5.14a	9.01ab	4.53b	9.22a	1.91c	2.04b	1.69c	2.30a
		T1	4.73a	7.27abc	3.04b	1.22b	2.69b	1.23c	1.31d	0.46b
		T2	2.99b	4.77bc	1.94c	2.15b	3.01a	1.74b	1.06d	0.49b

Control (CK): no N fertilizer (0 g/plant), medium (T1): 4 g/plant of pure N, and high (T2): 8 g/plant of pure N. Different lowercase letters within a column show the significant difference among treatments according to the least significant difference test (LSD; p < 0.05). N_in-SDS_, SDS-insoluble nitrogen; N_s_, SDS-soluble nitrogen; N_w_, water-soluble nitrogen; and N_np_, non-protein nitrogen.

### 3.5 Variations in the ratios of different nitrogen morphologies in the stem at various growth stages of tobacco plants under different treatments

The influence of N application, growth stage, and cultivar on the distribution of nitrogen morphologies within tobacco stems was apparent (**Table 4**). The analysis revealed a pronounced decline in the proportions of *N*_np_, *N*_w_, and *N*_s_ with increasing N application levels. Similarly, the ratio of *N*_np_ in the stems consistently decreased as the tobacco plants developed. Significant disparities between Hongda and K326 were noted, with Hongda demonstrating greater ratios of *N*_w_ and *N*_s_ in stems but lower proportions of *N*_np_ than K326.

**Table 4:** Allocation ratios of different nitrogen morphologies in stems at different growth stages and under various treatments of tobacco plants.

Days after transplanting	Cultivars	Treatments	2021				2022			
			*N*_in-SDS_ (%)	*N*_w_ (%)	*N*_s_ (%)	*N*_np_ (%)	*N*_in-SDS_ (%)	*N*_w_ (%)	*N*_s_ (%)	*N*_np_ (%)
25	Hongda	CK	0.83a	1.66ab	0.73a	0.34c	0.38d	6.24b	4.46b	0.39d
		T1	0.78ab	1.96a	0.94a	0.38c	0.48cd	8.23a	3.46c	0.52d
		T2	0.38c	1.02c	0.30b	1.22b	2.18b	4.55c	5.54a	0.81cd
	K326	CK	0.79ab	1.45bc	0.58ab	2.20a	0.69c	2.65d	1.53e	4.29a
		T1	0.92a	1.66ab	0.63ab	1.13b	0.40d	4.26c	1.42e	3.25b
		T2	0.54bc	1.10c	0.29b	2.29a	2.53a	1.33e	2.85d	1.27c
50	Hongda	CK	2.70a	6.58a	1.86ab	0.68d	1.00c	13.83a	7.18b	4.53b
		T1	1.98ab	6.25a	2.31a	5.36b	0.71c	15.56a	6.92b	3.04b
		T2	0.48c	1.49c	0.34c	7.55a	2.25b	10.88b	11.17a	3.16b
	K326	CK	2.19a	4.37b	1.74ab	3.51c	0.77c	4.83d	3.58c	16.16a
		T1	0.98bc	2.42c	0.83bc	6.45ab	0.40c	9.04bc	3.04c	7.49b
		T2	1.05bc	2.30c	0.87bc	6.23b	5.04a	7.55cd	7.06b	3.53b
75	Hongda	CK	0.71b	6.45a	2.24a	10.62b	5.87a	10.57a	3.44ab	2.80c
		T1	0.47b	3.24bc	1.22b	13.54a	4.86ab	12.71a	5.23a	9.19b
		T2	0.63b	1.39d	0.62cd	4.45c	3.72ab	12.87a	4.58a	14.69ab
	K326	CK	1.21a	4.71b	1.44b	10.42b	2.23b	3.01b	1.42c	18.12a
		T1	0.85b	1.39d	0.48d	14.48a	1.99b	5.87b	2.17bc	13.75ab
		T2	0.58b	2.61cd	0.80c	2.19d	1.66b	6.04b	2.02bc	19.92a
100	Hongda	CK	2.43a	7.69a	3.41a	11.24c	4.13bc	7.90b	4.46b	9.32c
		T1	1.23bc	3.71b	1.44c	13.55bc	6.02a	15.87a	5.54b	3.75d
		T2	0.49c	2.96b	1.02c	15.87ab	4.07bc	15.36a	7.35a	8.70c
	K326	CK	1.93ab	5.87ab	2.48b	16.58ab	2.34d	2.92c	2.27c	18.76a
		T1	0.46c	2.87b	1.03c	17.68a	2.68cd	6.77b	2.62c	15.42ab
		T2	0.56c	3.01b	0.95c	16.13ab	4.20b	9.50b	4.87b	13.09b

Control (CK): no N fertilizer (0 g/plant), medium (T1): 4 g/plant of pure N, and high (T2): 8 g/plant of pure N. Different lowercase letters within a column show the significant difference among treatments according to the least significant difference test (LSD; p < 0.05). N_in-SDS_, SDS-insoluble nitrogen; N_s_, SDS-soluble nitrogen; N_w_, water-soluble nitrogen; and N_np_, non-protein nitrogen.

### 3.6 Nitrogen distribution influences nitrogen accumulation in tobacco plants

**Figure 3:**
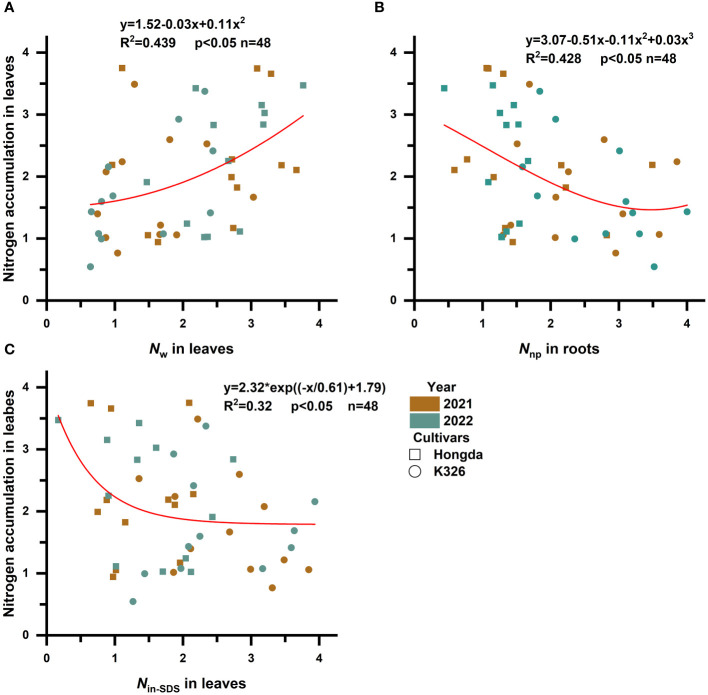
Scatterplot with fit curve illustrates the effects of different distributions of nitrogen morphologies on leaf nitrogen accumulation (*Z* score). *N*_in-SDS_, SDS-insoluble nitrogen; *N*_s_, SDS-soluble nitrogen; *N*_w_, water-soluble nitrogen; and *N*_np_, non-protein nitrogen. **(A–C)** represent the relationship between *N*_w_ in leaves, *N*_np_ in roots, and *N*_in-SDS_ in leaves in relation to the nitrogen accumulation of the leaves, respectively.

To explore how various N morphologies affect N and biomass accumulation in flue-cured tobacco, we conducted a standardized data analysis using the *Z* score method (**Supplementary Tables 1–4**). A subsequent investigation into the influence of different N morphologies on N accumulation in flue-cured tobacco leaves (**Figure 3**; **Table 5**) indicated that increased proportions of *N*_w_ in leaves were associated with an increase in overall N accumulation. Moreover, greater proportions of *N*_in-SDS_ in leaves and *N*_np_ in roots are linked to a reduction in overall N accumulation. A quadratic function accurately described the relationship between *N*_w_ and N accumulation in leaves, shedding light on the intricate dynamics. Notably, cubic curves accurately represented the relationship between *N*_np_ and N accumulation in roots, revealing a complex and nonlinear association between these factors. Similarly, the correlation between *N*_in-SDS_ in leaves and N accumulation was effectively characterized by an exponential function, highlighting the intricate and nonlinear interplay between these variables. These findings underscore the multifaceted interactions between different N morphologies and biomass accumulation in flue-cured tobacco leaves, providing valuable insights into the underlying mechanisms that influence crop growth and development.

**Table 5:** Curve fitting analysis illustrates the effects of the distributions of different nitrogen morphologies on nitrogen accumulation in leaves ((Z score+2).).

Independent variable	Mode	Relationship	R^2^	p	n
*N*_w_ in leaves	Quadratic	*y* = 1.52 − 0.03*x* + 0.11*x* ^2^	0.439	p < 0.05	48
*N*_np_ in roots	Cubic	*y* = 3.07 − 0.51*x* − 0.11*x* ^2^ + 0.03*x* ^3^	0.428	p < 0.05	48
*N*_in-SDS_ in leaves	Exponential	*y* = 2.32*exp[(−*x*/0.61) + 1.79]	0.320	p < 0.05	48

N_in-SDS_, SDS-insoluble nitrogen; N_w_, water-soluble nitrogen; and N_np_, non-protein nitrogen.

### 3.7 Effect of N distribution on biomass accumulation in tobacco plants

**Figure 4:**
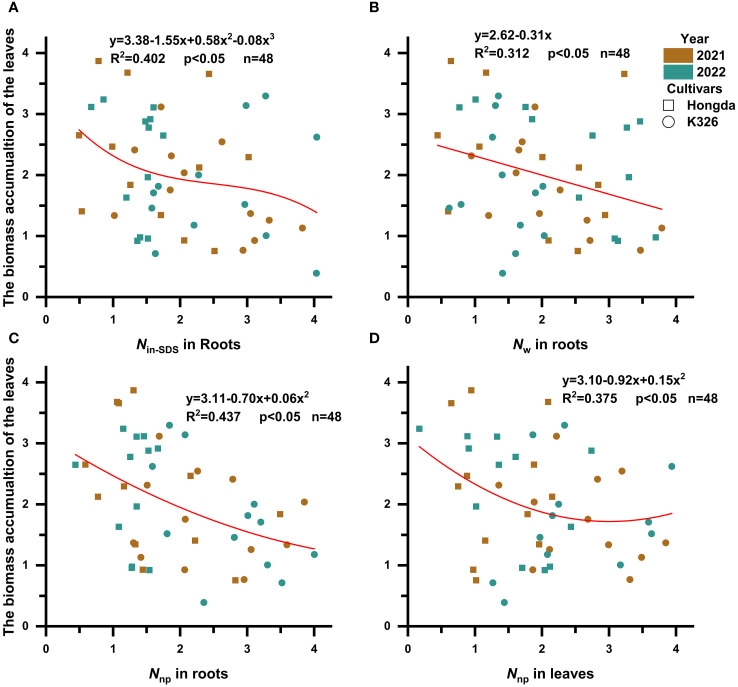
Scatterplot with fit curve illustrates the effects of the distributions of different nitrogen morphologies (*Z* score) on leaf biomass accumulation (*Z* score). *N*_in-SDS_, SDS-insoluble nitrogen; *N*_w_, water-soluble nitrogen; *N*_np_, non-protein nitrogen. **(A–D)** represent the relationship between *N*_in-SDS_ in roots, *N*_w_ in roots, *N*_np_ in roots, and *N*_np_ in leaves in relation to the biomass accumulation of the leaves, respectively.

It became apparent that the presence of *N*_in-SDS_, *N*_w_, and *N*_np_ in roots and *N*_np_ in leaves hindered the accumulation of biomass in tobacco leaves (**Figure 4**; **Table 6**). Notably, the inhibitory effects of *N*_in-SDS_ and *N*_np_ on roots and *N*_np_ on leaves did not follow a linear pattern despite their increasing proportions. Specifically, the relationship between *N*_in-SDS_ in roots and biomass accumulation in leaves can be described as a cubic function, highlighting the intricate interplay between these variables. Similarly, the relationship between *N*_w_ in roots and N accumulation in leaves followed a linear function. Additionally, the association between *N*_np_ in roots and biomass accumulation in leaves can be described as a quadratic function, suggesting a complex interplay between these variables. Finally, the relationship between *N*_np_ and biomass accumulation in leaves can be described as a quadratic function, indicating a complex and nonlinear association between these factors. These functional relationships provide valuable insights into the dynamics of biomass distribution and accumulation in flue-cured tobacco leaves.

**Table 6:** Curve fitting analysis illustrates the effects of the distributions of different nitrogen morphologies (Z score+2) on leaf biomass production (Z score+2).

Independent variable	Mode	Relationship	R^2^	p	n
*N*_in-SDS_ in roots	Cubic	*y* = 3.38 − 1.55*x* + 0.58*x* ^2^ − 0.08*x* ^3^	0.402	p < 0.05	48
*N*_w_ in roots	Linear	*y* = 2.62 − 0.31*x*	0.312	p < 0.05	48
*N*_np_ in roots	Quadratic	*y* = 3.11 − 0.70*x*+ 0.06*x* ^2^	0.437	p < 0.05	48
*N*_np_ in leaves	Quadratic	*y* = 3.10 − 0.92*x* + 0.15*x* ^2^	0.375	p < 0.05	48

N_in-SDS_, SDS-insoluble nitrogen; N_w_, water-soluble nitrogen; and N_np_, non-protein nitrogen.

## 4 Discussion

Nitrogen (N) is a critical factor for the growth and development of plants and influences various metabolic functions and cellular processes that ultimately determine crop yield (Song et al., 2022). Despite the fact that extensive studies demonstrated the role of N in plants and its impact on plant growth (Moose and Below, 2009), the specific distribution of N morphologies across different plant organs and their influence on N and biomass accumulation under varying N application levels, growth stages, and cultivars remain poorly understood. Thus, the present study aimed to investigate the effects of various N fertilizer applications on two distinct cultivars, highlighting their varied N and biomass accumulation capabilities at different growth stages over 2 years (2021 and 2022).

Liu et al. (2018) and Ghafoor et al. (2021) emphasized the profound impact of N fertilizer application on N and biomass accumulation in tobacco leaves. Our study revealed that treatments T1 and T2 significantly increased N and biomass accumulation in both cultivars compared to those in plants without N fertilizer treatment (CK). Specifically, compared with the CK, treatments T1 and T2 resulted in notable increases in N accumulation of 58.03% and 118.97%, respectively. Similarly, T1 and T2 significantly enhanced biomass accumulation by 77.59% and 126.86%, respectively, indicating the positive influence of N application on tobacco growth. The findings suggested that the biomass and N accumulation followed the “S model” (Yang et al., 2012). The N and biomass accumulation rate initially increased and then decreased. Under CK, compared with those under T1 and T2, an increasing trend were observed at 50 d after transplanting, possibly due to the inhibition resulting from a high-N environment (Xun et al., 2020; Wang et al., 2022a). However, after 75 d, increased N application led to greater N and biomass accumulation rates, ultimately resulting in greater N and biomass accumulation at 125 d after transplanting. Previous studies have emphasized significant differences in NUE among flue-cured tobacco cultivars, which are attributable to variations in their ability to absorb N and accumulate biomass under similar environmental conditions. In earlier studies, Hongda displayed a greater NUE than K326 (Parker, 2008; Liang et al., 2013; Fan et al., 2018). Compared to the K326 cultivar, the Hongda cultivar consistently exhibited enhanced N and biomass accumulation, with increases of 16.18% and 19.83%, respectively, under various treatment conditions. The superior efficiency of Hongda is consistent with earlier research findings. This study monitored the increase in N and biomass in the whole plant and its leaves every 25 d compared to the corresponding periods under CK. Hongda accumulated more biomass and N in the whole plant and leaves 0–50 days after transplanting. Moreover, Hongda plants accumulated more biomass and N under the various N fertilizer treatments at 125 d after transplanting. These results indicate that Hongda possesses greater biomass and N accumulation abilities than K326, which is consistent with previous studies (Parker, 2008; Liang et al., 2013).

In our investigation, *N*_w_ (17.5%–32.7% of the total N in the plant) was the predominant morphology in leaves (**Supplementary Figure 1A**), which is consistent with earlier findings (Niinemets and Tenhunen, 1997; Takashima et al., 2004; Liu et al., 2018). *N*_w_ was found to be related to plant respiration and carboxylation. Similarly, *N*_s_ is associated with electron transport and light capture systems, accounting for 12.9%–19.6% of the total N in the whole plant, a ratio less than that of *N*_w_, which is also in line with earlier studies (Niinemets and Tenhunen, 1997; Takashima et al., 2004; Liu et al., 2018). The proportions of *N*_in-SDS_ and *N*_np_ were lower than those of *N*_s_ and *N*_w_, and these morphologies are primarily associated with plant structural and storage functions (Liu et al., 2018). We found that stems exhibited lower N allocation compared to leaves (**Supplementary Figure 1B**). This distinction can be attributed to the relatively weaker respiration and photosynthetic activity observed in stems than in leaves, as previously suggested (Ávila-Lovera et al., 2017; Tokarz et al., 2021; Salomón et al., 2022). Consequently, noticeable reductions in *N*_w_ and *N_s_* levels within the stems were observed. Remarkably, stems contain more *N*_np_ than other N morphologies, and *N*_np_ was also recognized for its significant role in plant development (Liu et al., 2018). Moreover, Fritschi et al. (2013) explained that 73%–80% of the N in the roots of soybeans was mobile, which was closely associated with yield. In our study, the mobile N highlighted by Fritschi et al. (2013) may correspond to the *N*_np_ form of N.

Compared to the N distribution in leaves, a greater proportion of N was allocated to *N*_w_ and *N*_np_ in roots (**Supplementary Figure 1C**). *N*_w_ in roots is mostly associated with root respiration, a critical physiological trait that contributes to root resource acquisition strategies. Similarly, root respiration, a fundamental metabolic process responsible for growth, ion mobilization and uptake, and cell maintenance through ATP production, carbon skeleton production, and redox balancing, is closely related to N absorption and biomass accumulation (Han and Zhu, 2021). Similar to *N*_np_ in stems, *N*_np_ in roots also plays a role in remobilizing N, contributing to plant growth (Fritschi et al., 2013; Liu et al., 2018). Among the different treatments, the ratios of *N*_w_, *N*_s_, and *N*_np_ in the tobacco roots decreased with increasing N application, suggesting a decrease in root respiration and storage functions, resulting in a failure to provide sufficient energy for N uptake and biomass accumulation (Han and Zhu, 2021). *N*_np_ in stems also decreased with increasing N application, indicating insufficient N sources available for plant growth (Ávila-Lovera et al., 2017). In leaves, *N*_in-SDS_ decreased with increasing N application, whereas *N*_w_ and *N*_s_ increased with increasing N application, indicating that N application can promote respiration and photosynthesis and facilitate N storage for leaf expansion (Liu et al., 2018).

The distribution of N may account for the observed variation in N uptake and biomass accumulation at different growth stages. During the growth of tobacco plants, the proportion of *N*_in-SDS_ in leaves decreased, whereas that of *N*_s_ and *N*_np_ increased. Initially, a greater allocation of *N*_in-SDS_ to the leaves may facilitate the development of larger leaves, as suggested by Liu et al. (2018). As the plants matured, the increased *N*_s_ encouraged robust photosynthesis, whereas elevated *N*_np_ supported leaf expansion, consistent with prior research indicating rapid early-stage leaf growth, followed by deceleration in later stages (Hernández-Montes et al., 2019; He and Qin, 2020). Concurrently, the proportions of *N*_in-SDS_ and *N*_np_ in the stems decreased as the tobacco plants continued to develop. Initially, a greater *N*_in-SDS_ allocation bolstered the growth of sturdy stems, which is beneficial for overall plant development (Guo et al., 2018). Subsequently, the reduced distribution of *N*_np_ in stems implies a diminished capacity for stem growth, with the utilized N contributing less to storage and promoting plant growth through remobilized N, which is consistent with the findings of Darenova et al. (2020) and Liu et al. (2018). As the tobacco plants developed, the proportion of *N*_in-SDS_ in roots decreased. Higher *N*_in-SDS_ allocation helped tobacco establish stronger roots in the early stages. Over time, the reduced *N*_in-SDS_ distribution in roots indicated a weakened ability of roots to grow and gradually stop, with N being used for respiration, thereby promoting N uptake (Jiang et al., 2023). Plants obtained more vital nutrient absorption abilities in the later stages than in the earlier stages (Khan et al., 2020).

Nitrogen distribution may explain the differences in N uptake and biomass accumulation among different cultivars (Li et al., 2023). The ratio of *N*_w_ and *N*_s_ in the stems and leaves of the whole plant of Hongda was greater than that in the stems and leaves of the whole plant of K326, suggesting that Hongda had greater respiration and more N sources for stem and leaf growth, contributing to different abilities of N uptake and use (Pan et al., 2016; An et al., 2019). By curve fitting analysis, we determined that *N*_in-SDS_, *N*_w_, and *N*_np_ in roots and *N*_np_ in leaves were the major factors contributing to biomass accumulation in leaves. *N*_w_ in leaves, *N*_np_ in roots, and *N*_in-SDS_ in leaves were the main factors influencing N accumulation, ultimately determining the differences in N uptake and biomass accumulation abilities among different N applications, stages, and cultivars. *N*_w_ in leaves, *N*_np_ in roots, and *N*_in-SDS_ in leaves were the primary factors influencing N accumulation. With increasing N fertilizer application, *N*_w_ in leaves increases, which enhances carboxylation and respiration and directly provides more energy for N absorption (Khan et al., 2022). The proportion of *N*_in-SDS_ in leaves decreased with tobacco growth, promoting N uptake and accelerating N accumulation 75 days after transplanting. Hongda plants had a greater percentage of *N*_w_ in their leaves than K326 plants, which improved their photosynthetic ability and promoted N uptake (Balestrini et al., 2020; Su et al., 2020). The distribution of *N*_w_ in leaves primarily affected N accumulation at different N fertilizer application levels. In contrast, *N*_in-SDS_ in leaves mainly influenced N accumulation in tobacco leaves at different growth stages. Moreover, *N*_w_ in leaves primarily affects the N accumulation in different tobacco cultivars. Overall, these N morphologies collectively coordinate N accumulation in tobacco leaves.

Increases in *N*_in-SDS_, *N*_w_, and *N*_np_ in roots and *N*_np_ in leaves were found to adversely affect biomass accumulation. Although increased N fertilizer application has exhibited adverse effects on the ratio of *N*_w_ and *N*_np_ in roots, inhibiting root respiration and development (Liu et al., 2018), under an abundant N environment, decreased respiration in roots results in decreased organic carbon compound consumption, which can promote biomass accumulation in tobacco leaves (Weitz et al., 2021). In contrast, *N*_np_ in leaves also increases with increasing N fertilizer application, which decreases the ratio of respiration and photosynthesis abilities and adversely affects leaf development (Niinemets and Tenhunen, 1997; Takashima et al., 2004; Liu et al., 2018). Among the different stages, *N*_np_ in leaves increased with tobacco growth during the various stages, in contrast to the results of Liu et al. (2018), which might be attributed to the focus on plant growth under low-N environments. In contrast, our study examined N allocation under different N conditions. The ratio of *N*_np_ in leaves increased at different stages, adversely affecting leaf growth; however, the ratio of *N*_in-SDS_ in roots decreased with tobacco growth, promoting leaf biomass accumulation, indicating that *N*_np_ in leaves is more closely related to leaf biomass accumulation (Liu et al., 2018). Between Hongda and K326, *N*_in-SDS_ and *N*_np_ in roots and *N*_np_ in leaves were lower in Hongda than in K326, thereby enhancing leaf growth under similar treatment conditions (An et al., 2019). The allocation of *N*_w_ in roots and *N*_np_ in leaves primarily influenced the differences in biomass accumulation among the N application levels. *N*_np_ in leaves primarily affects the disparities in biomass accumulation at different growth stages. Additionally, *N*_in-SDS_ and *N*_np_ in roots and *N*_np_ in leaves contributed to determining the differences in biomass accumulation among the varieties.

## 5 Conclusions

Our study demonstrated significant increases in nitrogen and biomass accumulation in treatments T1 and T2 as compared to control (CK). Across various experimental conditions and growth stages, the Hongda cultivar consistently exhibited greater nitrogen and biomass accumulation than the K326 cultivar. The allocation ratios of nitrogen morphologies profoundly influence vital physiological processes such as photosynthesis and respiration, improve nitrogen absorption, and promote optimal plant growth. In response to diverse nitrogen conditions, tobacco plants adapt to these allocation ratios of nitrogen morphologies to efficiently absorb and utilize nitrogen. Hongda showed superior nitrogen use efficiency to K326, which was credited to its scientific honing strategy of allocating nitrogen. Moreover, these allocation ratios play a pivotal role in aiding nitrogen uptake and utilization and facilitating overall plant development across various growth stages. Future studies should focus on investigating the molecular mechanisms underlying nitrogen partitioning to enhance nitrogen use efficiency and plant productivity.

## Data availability statement

The original contributions presented in the study are included in the article/**Supplementary Material**. Further inquiries can be directed to the corresponding authors.

## Author contributions

SL: Conceptualization, Data curation, Investigation, Methodology, Software, Validation, Writing – original draft, Writing – review & editing. WA: Conceptualization, Data curation, Methodology, Software, Writing – original draft, Writing – review & editing. TJ: Data curation, Methodology, Software, Writing – original draft. YY: Writing – original draft, Data curation, Formal analysis. LY: Data curation, Formal analysis, Methodology, Writing – original draft. TZ: Data curation, Investigation, Software, Writing – original draft. FM: Formal analysis, Software, Validation, Writing – original draft. SAA: Writing – original, Writing - review & editing. QS: Conceptualization, Data curation, Methodology, Writing – original draft. CG: Conceptualization, Data curation, Software, Writing – original draft. ZZ: Conceptualization, Funding acquisition, Project administration, Resources, Supervision, Writing – original draft, Writing – review & editing.
