# Supplementary material for: Deciphering the impact of nitrogen morphologies distribution on nitrogen and biomass accumulation in tobacco plants
Source: Front Plant Sci. 2024 Jul 1;15:1377364. doi: 10.3389/fpls.2024.1377364 (PMC11246850; doi:10.3389/fpls.2024.1377364)
Supplement: Supplementary file 1 [file DataSheet_1.docx]

**Deciphering the impact of nitrogen morphologies distribution on nitrogen and biomass accumulation in tobacco plants**

Shichen Li^a,†^, Tao Jiang^a,†^, Waqar Ahmed^a,^*, Yingfen Yang^a^, Linyuan Yang^a^, Tao Zhang^a^, Fupeng Mei^a^, Sulaiman Ali Alharbi^b^, Qu Shan^a^, Cuilian Guo^a^, and Zhengxiong Zhao^a,^*

^a^Yunnan Agricultural University, Kunming 650201, Yunnan, China.

^b^Department of Botany & Microbiology College of Science, King Saud University, Riyadh 11451, Saudi Arabia.

† Authors contributed equally to this work and shared first authorship.

**Corresponding author:**

**Waqar Ahmed**

**E-mail: ahmed.waqar1083@yahoo.com**

**Zhengxiong Zhao**

**E-mail:** [**zhaozx0801@163.com**](mailto:zhaozx0801@163.com)

| **Table S1.** Nitrogen accumulation in leaves (Z-score +2) at different growth stages intervals post-transplantation in 2021 and 2022 of tobacco plants | | | | | | |
| --- | --- | --- | --- | --- | --- | --- |
| Year | Cultivars | Treatments | Days after transplanting | | |  |
|  |  |  | 25- 50d | 50- 75d | 75d- 100d | 100- 125d |
| 2021 | Hongda | CK | 3.74 | 0.94 | 1.05 | 2.19 |
|  |  | T1 | 2.28 | 2.18 | 1.99 | 1.17 |
|  |  | T2 | 1.82 | 3.75 | 2.11 | 3.66 |
|  | K326 | CK | 2.08 | 1.22 | 0.77 | 1.4 |
|  |  | T1 | 1.07 | 1.67 | 2.6 | 1.06 |
|  |  | T2 | 1.01 | 2.24 | 3.49 | 2.53 |
| 2022 | Hongda | CK | 2.84 | 1.03 | 1.02 | 1.24 |
|  |  | T1 | 2.25 | 3.03 | 1.91 | 1.11 |
|  |  | T2 | 3.42 | 2.83 | 3.47 | 3.15 |
|  | K326 | CK | 1.41 | 0.55 | 1.08 | 1.43 |
|  |  | T1 | 1.08 | 2.41 | 1.6 | 1.69 |
|  |  | T2 | 0.99 | 2.16 | 2.92 | 3.37 |
| **Here:** CK; No nitrogen fertilizer as control, T1; 4 g of pure nitrogen (medium)/plant, and T2; 8 g of pure nitrogen (high)/plant. The significant difference among treatments is shown by different lowercase letters within a column according to the least significant difference test (LSD; p < 0.05). | | | | | | |

| **Table S2.** Biomass accumulation in leaves (Z-score +2) at different growth stages intervals post-transplantation in 2021 and 2022 of tobacco plants | | | | | | |
| --- | --- | --- | --- | --- | --- | --- |
| Year | Cultivars | Treatments | Days after transplanting | | |  |
|  |  |  | 25- 50d | 50- 75d | 75d- 100d | 100- 125d |
| 2021 | Hongda | CK | 3.66 | 0.93 | 0.76 | 1.84 |
|  |  | T1 | 2.12 | 2.47 | 2.29 | 1.34 |
|  |  | T2 | 1.41 | 3.68 | 2.65 | 3.87 |
|  | K326 | CK | 2.54 | 1.13 | 0.77 | 1.26 |
|  |  | T1 | 1.34 | 1.76 | 2.41 | 1.37 |
|  |  | T2 | 0.93 | 2.04 | 3.12 | 2.31 |
| 2022 | Hongda | CK | 2.88 | 0.96 | 0.98 | 0.92 |
|  |  | T1 | 2.92 | 2.78 | 1.63 | 1.97 |
|  |  | T2 | 2.65 | 3.11 | 3.24 | 3.12 |
|  | K326 | CK | 1.71 | 0.71 | 1.01 | 1.18 |
|  |  | T1 | 1.46 | 1.82 | 2 | 1.52 |
|  |  | T2 | 0.39 | 2.62 | 3.14 | 3.3 |
| **Here:** CK; No nitrogen fertilizer as control, T1; 4 g of pure nitrogen (medium)/plant, and T2; 8 g of pure nitrogen (high)/plant. The significant difference among treatments is shown by different lowercase letters within a column according to the least significant difference test (LSD; p < 0.05). | | | | | | |

| **Table S3.** The ratios of nitrogen morphologies in leaves, stems, and roots of the nitrogen accumulation of the whole plant at different growth stages and under various treatments in 2021 (Z-score +2) | | | | | | | | | | | | | | |
| --- | --- | --- | --- | --- | --- | --- | --- | --- | --- | --- | --- | --- | --- | --- |
| Days after transplanting | Cultivars | Treatments | Roots | | | | Stems | | | | Leaves | | | |
|  |  |  | *N_in-SDS_* | *N_w_* | *N_s_* | *N_np_* | *N_in-SDS_* | *N_w_* | *N_s_* | *N_np_* | *N_in-SDS_* | *N_w_* | *N_s_* | *N_np_* |
| 25 | Hongda | CK | 2.43 | 3.23 | 3.6 | 1.08 | 2.61 | 2.51 | 2.6 | 0.91 | 0.65 | 3.09 | 2.14 | 1.28 |
|  |  | T1 | 2.28 | 2.55 | 2.3 | 0.77 | 2.36 | 3.34 | 3.44 | 0.96 | 2.15 | 2.72 | 3.66 | 0.77 |
|  |  | T2 | 0.53 | 0.6 | 0.65 | 2.22 | 0.4 | 0.74 | 0.89 | 1.95 | 1.15 | 2.79 | 2.4 | 1.54 |
|  | K326 | CK | 2.62 | 1.7 | 1.58 | 2.26 | 2.41 | 1.93 | 2.01 | 3.11 | 3.19 | 0.87 | 0.89 | 3.02 |
|  |  | T1 | 1.02 | 1.2 | 1.53 | 3.59 | 3.05 | 2.51 | 2.21 | 1.85 | 2.99 | 1.66 | 1.8 | 2.08 |
|  |  | T2 | 3.11 | 2.72 | 2.35 | 2.07 | 1.18 | 0.96 | 0.86 | 3.22 | 1.86 | 0.87 | 1.11 | 3.31 |
| 50 | Hongda | CK | 2.06 | 2.1 | 3.2 | 1.44 | 3.33 | 3.24 | 2.71 | 0.28 | 0.97 | 1.63 | 2.45 | 2.64 |
|  |  | T1 | 0.99 | 1.06 | 0.48 | 2.15 | 2.49 | 3.08 | 3.3 | 2.16 | 0.88 | 3.45 | 0.29 | 1.25 |
|  |  | T2 | 1.21 | 1.17 | 1.98 | 1.06 | 0.73 | 0.89 | 0.7 | 3.04 | 2.09 | 1.11 | 1.97 | 3.3 |
|  | K326 | CK | 3.82 | 3.79 | 2.93 | 1.41 | 2.73 | 2.22 | 2.55 | 1.42 | 3.48 | 1.67 | 3.26 | 1.12 |
|  |  | T1 | 1.85 | 2.27 | 2.03 | 2.08 | 1.32 | 1.32 | 1.34 | 2.6 | 2.69 | 3.03 | 1.64 | 0.98 |
|  |  | T2 | 2.06 | 1.61 | 1.38 | 3.85 | 1.4 | 1.26 | 1.4 | 2.51 | 1.88 | 1.11 | 2.39 | 2.71 |
| 75 | Hongda | CK | 2.51 | 2.53 | 3.31 | 2.82 | 1.88 | 3.59 | 3.7 | 2.27 | 1.02 | 1.48 | 2.06 | 1.78 |
|  |  | T1 | 3.02 | 2.01 | 2.27 | 1.16 | 0.97 | 1.97 | 2.13 | 2.86 | 0.75 | 2.71 | 3.71 | 1.16 |
|  |  | T2 | 0.49 | 0.44 | 0.34 | 0.59 | 1.57 | 1.04 | 1.21 | 1.02 | 1.88 | 3.66 | 1.85 | 2.58 |
|  | K326 | CK | 2.94 | 3.47 | 2.57 | 2.95 | 3.78 | 2.71 | 2.47 | 2.23 | 3.31 | 1.04 | 1.41 | 1.32 |
|  |  | T1 | 1.32 | 1.65 | 1.65 | 2.78 | 2.41 | 1.04 | 1 | 3.05 | 2.83 | 1.81 | 2.24 | 1.4 |
|  |  | T2 | 1.72 | 1.9 | 1.85 | 1.69 | 1.38 | 1.65 | 1.49 | 0.56 | 2.22 | 1.29 | 0.73 | 3.76 |
| 100 | Hongda | CK | 1.26 | 2.84 | 1.98 | 3.49 | 3.49 | 3.68 | 3.68 | 0.33 | 1.78 | 0.96 | 2.3 | 1.33 |
|  |  | T1 | 1.71 | 2.94 | 2.34 | 1.33 | 2.06 | 1.68 | 1.72 | 1.31 | 1.96 | 2.74 | 3.36 | 1.14 |
|  |  | T2 | 0.78 | 0.64 | 0.77 | 1.3 | 1.17 | 1.3 | 1.3 | 2.29 | 0.94 | 3.29 | 1.95 | 2.9 |
|  | K326 | CK | 3.33 | 2.67 | 3.61 | 3.06 | 2.89 | 2.76 | 2.75 | 2.6 | 2.11 | 0.75 | 0.33 | 1 |
|  |  | T1 | 3.05 | 1.96 | 2.18 | 1.3 | 1.14 | 1.26 | 1.31 | 3.06 | 3.85 | 1.91 | 1.66 | 2.23 |
|  |  | T2 | 1.87 | 0.95 | 1.12 | 1.51 | 1.26 | 1.33 | 1.23 | 2.41 | 1.36 | 2.35 | 2.39 | 3.4 |
| **Here:** CK; No nitrogen fertilizer as control, T1; 4 g of pure nitrogen (medium)/plant, and T2; 8 g of pure nitrogen (high)/plant. *N*_in-SDS_: sodium dodecyl sulfate-insoluble nitrogen; *N*_s_: sodium dodecyl sulfate soluble nitrogen; *N*_w_: water-soluble nitrogen; *N*_np_: non-protein nitrogen. | | | | | | | | | | | | | | |

| **Table S4.** The ratios of nitrogen morphologies in leaves, stems, and roots of the nitrogen accumulation of the whole plant at different growth stages and under various treatments in 2022 (Z-score +2) | | | | | | | | | | | | | | | |
| --- | --- | --- | --- | --- | --- | --- | --- | --- | --- | --- | --- | --- | --- | --- | --- |
| Days after transplanting | Cultivars | Treatments | Roots | | | | Stems | | | | Leaves | | | |  |
|  |  |  | *N_in-SDS_* | *N_w_* | *N_s_* | *N_np_* | *N_in-SDS_* | *N_w_* | *N_s_* | *N_np_* | *N_in-SDS_* | *N_w_* | *N_s_* | *N_np_* |  |
| 25 | Hongda | CK | 1.48 | 3.46 | 3.33 | 1.53 | 1.25 | 2.69 | 2.77 | 1.16 | 2.74 | 3.18 | 2.99 | 0.78 |  |
|  |  | T1 | 1.55 | 1.85 | 2.49 | 1.67 | 1.36 | 3.49 | 2.15 | 1.24 | 0.91 | 2.67 | 2.52 | 1.52 |  |
|  |  | T2 | 1.75 | 2.75 | 2.73 | 0.44 | 3.1 | 2 | 3.43 | 1.42 | 1.36 | 2.19 | 1.31 | 2.37 |  |
|  | K326 | CK | 1.6 | 1.9 | 1.58 | 3.2 | 1.57 | 1.23 | 0.97 | 3.56 | 3.59 | 2.4 | 2.64 | 1.29 |  |
|  |  | T1 | 1.58 | 0.62 | 0.93 | 2.81 | 1.27 | 1.89 | 0.9 | 2.92 | 1.97 | 0.76 | 2.22 | 2.49 |  |
|  |  | T2 | 4.03 | 1.41 | 0.94 | 2.35 | 3.45 | 0.7 | 1.78 | 1.7 | 1.44 | 0.8 | 0.32 | 3.55 |  |
| 50 | Hongda | CK | 1.52 | 3.08 | 3.38 | 1.28 | 1.61 | 2.89 | 2.23 | 1.65 | 1.7 | 2.36 | 2.1 | 1.53 |  |
|  |  | T1 | 1.53 | 3.27 | 2.16 | 1.26 | 1.44 | 3.32 | 2.15 | 1.36 | 1.61 | 3.2 | 2.7 | 0.9 |  |
|  |  | T2 | 1.6 | 0.77 | 2.54 | 1.35 | 2.32 | 2.15 | 3.59 | 1.38 | 1.33 | 2.45 | 3.55 | 1.4 |  |
|  | K326 | CK | 1.63 | 1.61 | 1.23 | 3.52 | 1.47 | 0.63 | 1.01 | 3.93 | 1.27 | 0.64 | 1.35 | 3.6 |  |
|  |  | T1 | 1.68 | 2.01 | 0.53 | 3.01 | 1.26 | 1.69 | 0.83 | 2.23 | 2.16 | 2.44 | 1.51 | 2.78 |  |
|  |  | T2 | 4.04 | 1.26 | 2.16 | 1.59 | 3.9 | 1.32 | 2.19 | 1.45 | 3.94 | 0.91 | 0.8 | 1.8 |  |
| 75 | Hongda | CK | 1.4 | 3.7 | 3.77 | 1.28 | 3.45 | 2.5 | 2.19 | 0.36 | 2.12 | 2.31 | 2.99 | 1.95 |  |
|  |  | T1 | 1.2 | 2.55 | 2.58 | 1.08 | 2.86 | 3.02 | 3.36 | 1.38 | 2.43 | 1.47 | 2.36 | 1.09 |  |
|  |  | T2 | 0.86 | 1.01 | 1.51 | 1.15 | 2.19 | 3.06 | 2.94 | 2.26 | 0.17 | 3.76 | 3.19 | 1.24 |  |
|  | K326 | CK | 3.28 | 2.03 | 1.44 | 3.3 | 1.32 | 0.66 | 0.87 | 2.81 | 3.17 | 1.71 | 1.35 | 1.5 |  |
|  |  | T1 | 2.27 | 1.41 | 1.59 | 3.11 | 1.18 | 1.36 | 1.36 | 2.11 | 2.25 | 0.81 | 1.41 | 3.77 |  |
|  |  | T2 | 2.98 | 1.31 | 1.1 | 2.07 | 0.99 | 1.4 | 1.27 | 3.09 | 1.86 | 1.94 | 0.69 | 2.45 |  |
| 100 | Hongda | CK | 1.36 | 3.13 | 2.83 | 1.54 | 2.17 | 1.64 | 1.97 | 1.59 | 2.04 | 2.06 | 2.1 | 2.56 |  |
|  |  | T1 | 1.52 | 3.29 | 3.56 | 1.35 | 3.61 | 3.22 | 2.54 | 0.55 | 1.02 | 2.84 | 2.06 | 2.24 |  |
|  |  | T2 | 0.67 | 1.75 | 1.16 | 1.46 | 2.12 | 3.11 | 3.5 | 1.48 | 0.89 | 3.16 | 3.89 | 1.03 |  |
|  | K326 | CK | 2.21 | 1.68 | 1.95 | 4 | 0.8 | 0.66 | 0.81 | 3.36 | 2.08 | 0.66 | 1.26 | 3.62 |  |
|  |  | T1 | 2.96 | 0.79 | 1.42 | 1.8 | 1.06 | 1.42 | 1 | 2.73 | 3.63 | 0.97 | 1.29 | 1.32 |  |
|  |  | T2 | 3.28 | 1.35 | 1.08 | 1.84 | 2.22 | 1.96 | 2.19 | 2.3 | 2.34 | 2.32 | 1.4 | 1.23 |  |
| **Here:** CK; No nitrogen fertilizer as control, T1; 4 g of pure nitrogen (medium)/plant, and T2; 8 g of pure nitrogen (high)/plant. *N*_in-SDS_: sodium dodecyl sulfate-insoluble nitrogen; *N*_s_: sodium dodecyl sulfate soluble nitrogen; *N*_w_: water-soluble nitrogen; *N*_np_: non-protein nitrogen. | | | | | | | | | | | | | | | |


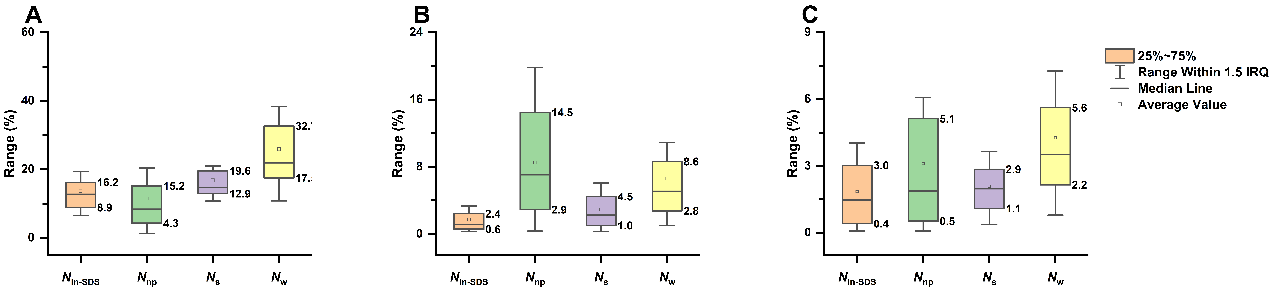


**Figure S1.** Tukey Test and boxplot of nitrogen morphologies distribution in each part of the nitrogen accumulation in the whole plant. *N*_in-SDS_: sodium dodecyl sulfate-insoluble nitrogen; *N*_s_: sodium dodecyl sulfate soluble nitrogen; *N*_w_: water-soluble nitrogen; *N*_np_: non-protein nitrogen. Panels (A), (B), (C) and (D) depict the ratio of nitrogen morphologies of the whole plant in leaf, stem and roots, respectively.
